# Is breast milk iodine concentration an influential factor in growth‐ and obesity‐related hormones and infants' growth parameters?

**DOI:** 10.1111/mcn.13078

**Published:** 2020-09-29

**Authors:** Pantea Nazeri, Zhale Tahmasebinejad, Mehdi Hedayati, Parvin Mirmiran, Fereidoun Azizi

**Affiliations:** ^1^ Family Health Institute, Breastfeeding Research Center Tehran University of Medical Sciences Tehran Iran; ^2^ Nutrition and Endocrine Research Center, Research Institute for Endocrine Sciences Shahid Beheshti University of Medical Sciences Tehran Iran; ^3^ Cellular and Molecular Endocrine Research Center, Research Institute for Endocrine Sciences Shahid Beheshti University of Medical Sciences Tehran Iran; ^4^ Department of Clinical Nutrition and Dietetics, Faculty of Nutrition Sciences and Food Technology, National Nutrition and Food Technology Research Institute Shahid Beheshti University of Medical Sciences Tehran Iran; ^5^ Endocrine Research Center, Research Institute for Endocrine Sciences Shahid Beheshti University of Medical Sciences Tehran Iran

**Keywords:** adiponectin, anthropometric measures, breast milk iodine concentration, infants, insulin‐like growth factor, leptin

## Abstract

Iodine, a key constituent of thyroid hormones, plays an indirect role in prenatal and postnatal growth. This study aimed to investigate whether breast milk iodine concentration (BMIC) is associated with growth‐ and obesity‐related hormones and subsequently the infants' anthropometric measures. In present study conducted in Tehran (Iran), 94 lactating mothers and healthy infants who were exclusively breastfed were included. Concentrations of iodine, insulin‐like growth factor‐1 (IGF‐1), adiponectin (AD) and leptin (LP) were measured in breast milk samples collected during 3‐ to 5‐day postpartum. Anthropometric measures of infants were assessed at 6 months of life, and age‐ and sex‐specific *z*‐score values were calculated using the World Health Organization growth standards. The median (interquartile range) iodine, IGF‐1, AD and LP concentrations were 232.5 (157.5–296.0) μg L^−1^, 15.7 (11.9–21.1) ng ml^−1^, 13.2 (5.1–29.8) mg L^−1^ and 1.16 (0.86–1.70) ng ml^−1^ in breast milk, respectively. No significant correlations were found between BMIC and IGF‐1, AD and LP concentrations during the first few days postpartum. In adjusted regression model, BMIC was positively associated with weight‐for‐length *z* score of infants. In the presence of IGF‐1, AD or LP, the coefficients of BMIC for weight‐for‐length *z* score of infants were *β* = .003 (*P* = .021), *β* = .002 (*P* = .028) or *β* = .003 (*P* = .013), respectively. No other anthropometric measurements were associated with iodine or growth‐ and obesity‐related hormones in breast milk. Our findings indicate that BMIC is a potential contributor to infants' growth status, independent of IGF‐1, AD or LP concentrations in breast milk. The underlying mechanisms remain to be elucidated.

**Key messages**

- The study adds important information to the sparse evidence on the associations between iodine and growth‐ and obesity‐related hormones in breast milk and subsequently infants' growth measurements.
- Breast milk iodine concentration during the early stage of lactation is positively associated with weight‐for‐length *z* scores in infants at 6 months of age, independent of IGF‐1, AD or LP concentrations in breast milk.
- The underlying mechanism of how iodine in breast milk affects infant growth status remains to be elucidated.

## 1 INTRODUCTION

Iodine requirements in infants are significantly higher than those in any other age groups because of the rapid thyroidal iodine turnover (Zimmermann, 2009). During lactation, the mammary gland concentrates iodine through an increased expression of the sodium/iodide symporter and secretes it into breast milk (Azizi & Smyth, 2009; Leung, Pearce, & Braverman, 2011). Hence, adequate breast milk iodine concentration (BMIC) is particularly important for breastfed infants, who are entirely dependent on iodine supplied via breast milk (Dold et al., 2017; Nazeri et al., 2018). It is well‐established that severe iodine deficiency during a critical period of rapid growth and brain development can lead to irreversible consequences, such as low intelligence, short stature, skeleton disorders and other growth retardation (Pearce, 2014; Zimmermann, Jooste, & Pandav, 2008).

Somatic growth from conception to adulthood is a complex process influenced by several direct and indirect factors. Iodine, a key constituent of thyroid hormones, is known to promote the synthesis and secretion of growth hormone (Zimmermann, 2011). The main function of growth hormone is to regulate the production of insulin‐like growth factors (IGFs), which enhance cell differentiation and proliferation (Hellström et al., 2016). Previous studies in school‐aged children have clearly shown that iodine repletion positively affects indirect markers of growth, specifically IGFs and their binding proteins (Zimmermann et al., 2007). However, definitive evidence stating that iodine repletion in pregnant women improves prenatal and postnatal growth outcomes is not available (Farebrother et al., 2018). Studies regarding the effects of iodine repletion during lactation on infants' growth parameters are limited (Bouhouch et al., 2014; Nazeri et al., 2018). Moreover, sufficient data to support the possible association between BMIC and infants' anthropometric measures do not exist.

It is well‐known that breast milk contains several bioactive components that are involved in the optimal growth and development of infants, including macro‐ and micronutrients, enzymes, cytokines, growth factors and hormones, such as IGFs, adiponectin (AD) and leptin (LP) (Bartok & Ventura, 2009; Young, Johnson, & Krebs, 2012). Findings regarding the association between breast milk AD and LP and growth in infancy are inconsistent (Fields, Schneider, & Pavela, 2016). Furthermore, the existing literature associating breast milk IGFs and infant outcomes is insufficient. It is unclear whether exposure to growth factors and hormones during the early stage of lactation, a period in which concentrations of these components are at their highest, determines subsequent growth status.

Some recent studies suggest that iodine might be involved in the regulation of obesity‐related hormones in breast milk. In a study conducted in lactating mothers, BMIC was inversely associated with AD levels (Velasco et al., 2016). A similar pattern was also observed in an in vitro study in which a potassium iodide dose similar to the BMIC significantly decreased the AD expression in human mature adipocytes (Gutierrez‐Repiso et al., 2014). These findings raise the question whether BMIC is an influential factor in growth‐ and obesity‐related hormones and infants' growth parameters. Therefore, the present study aimed to determine the concentration of iodine, IGF‐1, AD and LP in breast milk and their association during the early stage of lactation and to subsequently assess the associations between the aforementioned breast milk components and the anthropometric measures of infants at 6 months of life.

## 2 MATERIALS AND METHODS

### 2.1 Subjects

This study was conducted on a sample of our previous published study, which was originally designed to assess the effect of iodine supplementation during lactation period on maternal and infant iodine status during the first year of life (Nazeri, Tahmasebinejad, et al., 2018). Briefly, 180 lactating mother–neonate pairs referred to four healthcare centres of Tehran within 3‐ to 5‐day postpartum were randomly assigned to treatment groups (i.e., the placebo and 150 μg day^−1^ and 300 μg day^−1^ of iodine) in a 1:1:1 allocation ratio for 12 months. Considering that iodine supplementation had no effect on the infants' anthropometric measures, 94 lactating mothers with available breast milk samples during the first few days postpartum and healthy breastfed infants whose growth parameters were assessed at 6 months of life were included in the current study. The eligibility criteria were healthy mothers who had a singleton birth and intended to breastfeed exclusively and healthy infants born full term (gestational age, 37–42 weeks) with a normal birth weight (2,500–4,200 g) and were exclusively or partially breastfed at the first 6 months of life.

### 2.2 Anthropometric measures

Data on birth weight, length and head circumference were obtained from the infants' health records. At 6 months of life, the infants' anthropometric parameters were determined using calibrated healthcare centre equipment while infants were wearing light indoor clothing and with no shoes. The following measurements were obtained: weight (baby scale, precision of 100 g), length (length board, measured to the nearest 0.1) and head circumference (greatest circumference as above the eyebrows and pinna of the ears and around the occipital prominence at the back of the skull using a paper or metal tape). The software WHO Anthro (version 3.2.2, 2011, WHO, Geneva, Switzerland) was used to determine age‐ and sex‐specific *z*‐score values for weight‐for‐age, weight‐for‐length, length‐for‐age and head circumference‐for‐age.

### 2.3 Breast milk sample collection and laboratory measurements

At 3‐ to 5‐day postpartum before intervention initiation, mothers were provided labelled sterile plastic bottles (SUPA Medical Services, Tehran, Iran) to collect a 10‐ml sample of breast milk using manual expression or breast pump according to the given instructions. They were instructed to collect milk sample at the regular feeding time, usually 2 h after the previous nursing, and to store the milk sample in the refrigerator. All samples were collected and sent to the laboratory of the Research Institute for Endocrine Sciences, where they were transferred into screw‐top labelled plastic vials. The aliquots were kept frozen for up to 24 months at −80°C until analysis.

The iodine concentration of breast milk samples was analysed using the Sandell–Kolthoff (acid‐digestion) reaction. Intra‐assay coefficient variations (CVs) in BMIC values of 3.5, 12.7 and 36.2 μg dl^−1^ were 8.6%, 6.7% and 9.3%, respectively. The inter‐assay CVs at concentrations of 3.3, 12.9 and 35.7 μg dl^−1^ were 9.8%, 8.6% and 12.3%, respectively.

The total concentration of growth‐ and obesity‐related hormones was measured using a sandwich enzyme‐linked immunosorbent assay (ELISA) method for IGF‐1 (Mediagnost, Reutlingen, Germany), AD (ZellBio GmbH, Ulm, Germany) and LP (Mercodia, Uppsala, Sweden). The sensitivity and intra‐assay CVs were 0.09 ng ml^−1^ and 6.1% for IGF‐1, 0.1 mg L^−1^ and 4.9% for AD and 0.05 ng ml^−1^ and 5.2% for LP, respectively.

### 2.4 Statistical analysis

Frequency distribution (percentage), mean ± standard deviation (SD) and median (interquartile range [IQR]) were expressed for categorical and continuous variables. Normality of the variables was assessed using the Kolmogorov–Smirnov test and histogram chart. Breast milk iodine, IGF‐1, AD and LP concentrations were categorized into three groups. Kruskal–Wallis or analysis of variance (ANOVA) was used to assess significance of differences in growth‐ and obesity‐related hormones among tertiles of BMIC, as well as infant anthropometrics among tertiles of breast milk iodine, IGF‐1, AD and LP concentrations. The correlations between BMIC and growth‐ and obesity‐related hormones were assessed by Spearman's correlation coefficients. A multivariate multiple linear regression model was used to identify the associations between BMIC and each growth‐ and obesity‐related hormones, and infants' growth parameters and the coefficient and standard error were calculated. The multivariate term refers to the three dependent variables (weight‐for‐age or weight‐for‐length, length‐for‐age and head circumference‐for‐age) that were analysed jointly. Factors including maternal age, educational level, pre‐pregnancy maternal body mass index (BMI), parity, type of infant feeding at 6 months and anthropometric measures at birth were considered covariates. All statistical analyses were performed using R software (version 3.4.2, www.r-project.org). A two‐tailed *P* value < 0.05 was considered statistically significant.

### 2.5 Ethical considerations

After receiving sufficient explanation regarding the study's protocol and objectives, the infants' parents provided written informed consent for inclusion in the study. The study's protocol was approved by the ethics committee of Tehran University of Medical Sciences.

## 3 RESULTS

Basic characteristics of lactating mothers and infants are shown in Table 1. The mean ± *SD* maternal age was 28.8 ± 5.2 years; over half of the mothers had normal pre‐pregnancy BMI (55.6%), and over 60% of them were multiparous (67.0%) and underwent caesarean sections (62.4%). The mean ± *SD* birth weight, length and head circumference in infants were 3,362 ± 377 g, 50.2 ± 2.2 cm and 35.3 ± 1.3 cm, respectively. All infants were exclusively breastfed within the first few days of life.

**TABLE 1:** Basic characteristics of mothers and infants, *n* = 94

Characteristics	*n*	%	Mean	*SD*
*Mothers*				
Age (year)			28.8	5.2
18–34	83	88.3		
35–44	11	11.7		
Pre‐pregnancy body mass index (kg m^−2^)			24.9	3.6
Underweight	7	7.8		
Normal weight	50	55.6		
Overweight/obese	33	36.7		
Education degree				
Primary	34	36.2		
Secondary	42	44.7		
Higher	18	19.1		
Date of last pregnancy (year)			4.2	4.4
Gravidity				
Primigravidity	27	28.7		
Multigravidity	67	71.3		
Parity				
Primiparous	31	33.0		
Multiparous	63	67.0		
History of abortion				
No	77	81.9		
Yes	17	18.1		
Mode of delivery				
Natural vaginal delivery	35	37.6		
Caesarean section	58	62.4		
*Infants*				
Sex				
Male	51	54.3		
Female	43	45.7		
Birth weight (g)			3362.3	377.0
Birth length (cm)			50.2	2.2
Birth head circumference (cm)			35.3	1.3

**FIGURE 1:**
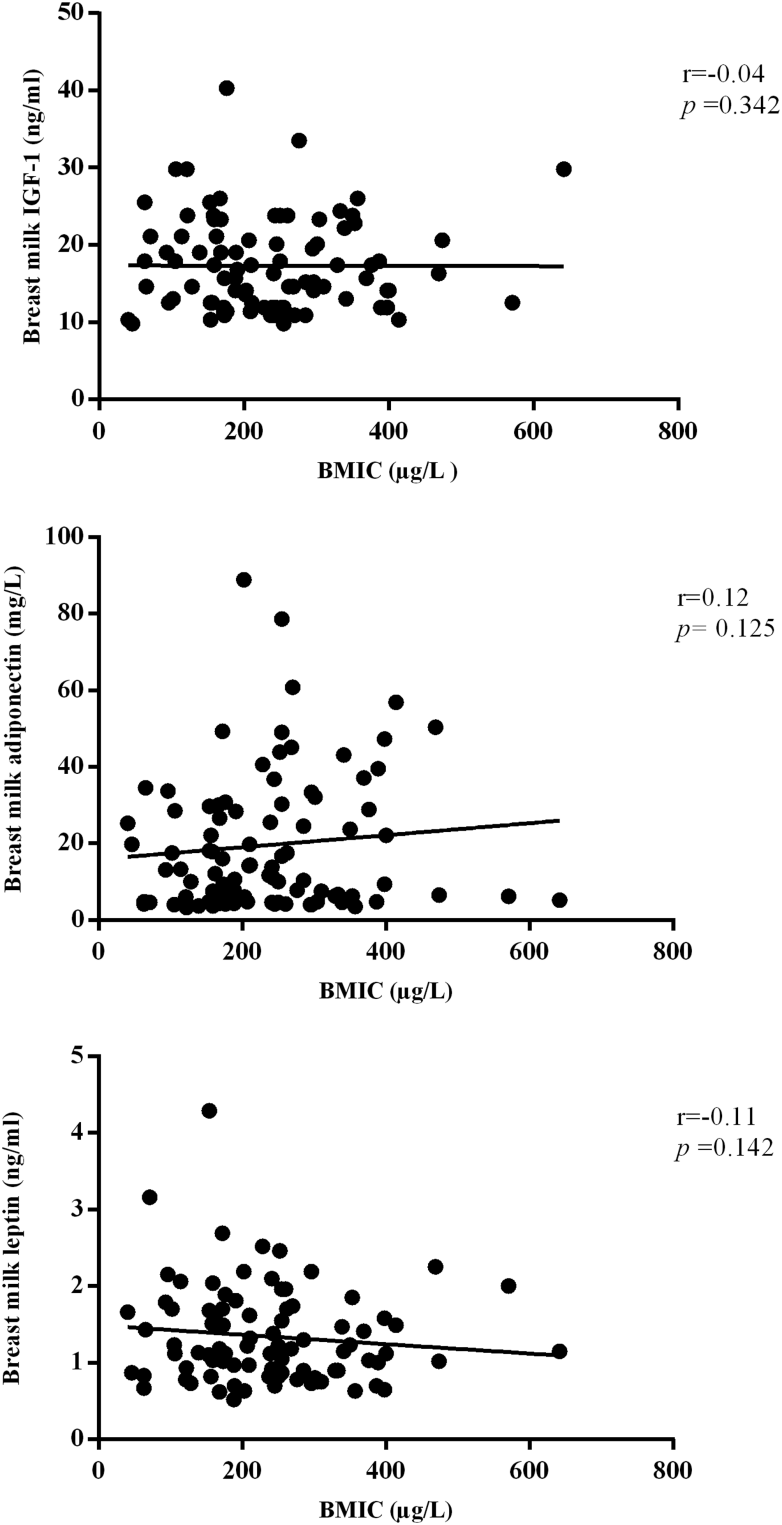
Correlations of breast milk iodine and IGF‐1, adiponectin and leptin concentrations during the early stage of lactation. BMIC, breast milk iodine concentration; IGF‐1, insulin‐like growth factor‐1

The concentrations of iodine, IGF‐1, AD and LP in breast milk samples collected during the first few days postpartum are presented in Table 2. Overall, the median (IQR) iodine, IGF‐1, AD and LP concentrations of breast milk were 232.5 (157.5–296.0) μg L^−1^, 15.7 (11.9–21.1) ng ml^−1^, 13.2 (5.1–29.8) mg L^−1^ and 1.16 (0.86–1.70) ng ml^−1^, respectively. When breast milk components were stratified into tertiles, the medians (IQRs) in the first versus third tertile were 133.5 (97.5–158.7) versus 341.0 (296.0–398.0) μg L^−1^ for iodine, 11.6 (10.9–12.1) versus 23.3 (20.6–25.5) ng ml^−1^ for IGF‐1, 4.6 (4.1–5.4) versus 35.6 (29.5–47.7) mg L^−1^ for AD and 0.78 (0.70–0.85) versus 1.92 (1.69–2.19) ng ml^−1^ for LP. There were no significant differences in IGF‐1, AD and LP concentrations across tertiles of breast milk iodine levels. As indicated in Figure 1, no significant correlations were observed between BMIC and IGF‐1 (*r* = 0.04, *P* = .342), AD (*r* = 0.12, *P* = .125) and LP (*r* = −0.11, *P* = .142). However, significant correlations between IGF‐1 and AD (*r* = −0.53, *P* = <.001), between AD and LP (*r* = 0.44, *P* < .001) and between IGF‐1 and LP (*r* = −0.21, *P* = .043) were observed.

**TABLE 2:** Components in breast milk samples collected during the first few days postpartum

	Breast milk components							
	Iodine (μg L^−1^)		IGF‐1 (ng ml^−1^)		Adiponectin (mg L^−1^)		Leptin (ng ml^−1^)	
**Median (IQR)**	232.5	(157.5–296.0)	15.7	(11.9–21.1)	13.2	(5.1–29.8)	1.16	(0.86–1.70)
**Range**	40.0–642.0		9.8–40.3		3.3–88.9		0.52–4.29	
**Median (IQR) by tertile**								
*Tertile 1*	133.5	(97.5–158.7)	11.6	(10.9–12.1)	4.6	(4.1–5.4)	0.78	(0.70–0.85)
*Tertile 2*	237.0	(191.0–250.0)	15.7	(14.6–17.4)	13.2	(9.8–17.6)	1.15	(1.05–1.32)
*Tertile 3*	341.0	(296.0–398.0)	23.3	(20.6–25.5)	35.6	(29.5–47.7)	1.92	(1.69–2.19)

Abbreviations: IGF‐1, insulin‐like growth factor‐1; IQR, interquartile range.

At 6 months of life, the mean ± *SD z* scores of weight‐for‐age, weight‐for‐length, length‐for‐age and head circumference‐for‐age of infants were 0.18 ± 0.90, −0.01 ± 1.10, 0.48 ± 1.06 and 0.19 ± 0.80, respectively. More than two‐third of the infants were exclusively breastfed at 6 months of life. There were no significant differences in *z*‐score values of weight‐for‐age, weight‐for‐length, length‐for‐age and head circumference‐for‐age of infants among the tertiles of breast milk components collected during the first few days of the postpartum. Moreover, Spearman's correlation coefficients did not show any correlations between the infants' anthropometric measures at 6 months of life and iodine, IGF‐1, AD and LP concentrations of breast milk (data not shown). Table 3 shows the associations between BMIC and each growth‐ and obesity‐related hormone and infants' anthropometric measures at 6 months of life using the multivariate multiple regression. In the model adjusting for maternal age, educational level, pre‐pregnancy maternal BMI, parity, type of infant feeding at 6 months and infants' anthropometric measures at birth, iodine concentration of breast milk was positively associated with weight‐for‐length *z* score of infants. In the presence of IGF‐1, AD or LP, the coefficients of BMIC for weight‐for‐length *z* score of infants were *β* = .003 (*P* = .021), *β* = .002 (*P* = .028) or *β* = .003 (*P* = .013), respectively. No other anthropometric measures were associated with iodine or growth‐ and obesity‐related hormones in breast milk.

**TABLE 3:** Association of breast milk iodine concentration and IGF‐I, adiponectin and leptin and anthropometric measures of infants at 6 months of life

	Weight‐for‐age			Weight‐for‐length			Length‐for‐age			Head circumference‐for‐age		
	*β*	(SE)	*P*	*β*	(SE)	*P*	*β*	(SE)	*P*	*β*	(SE)	*P*
A												
BMIC (μg L^−1^)	.001	(0.001)	.128	.003	(0.001)	.021	−.0005	(0.001)	.687	−.0004	(0.0007)	.613
IGF‐I (ng ml^−1^)	−.015	(0.015)	.317	−.034	(0.020)	.101	.025	(0.020)	.219	.011	(0.013)	.381
B												
BMIC (μg L^−1^)	.001	(0.001)	.151	.002	(0.001)	.028	−.0005	(0.001)	.696	−.0003	(0.0007)	.641
Adiponectin (mg L^−1^)	.008	(0.006)	.175	.009	(0.008)	.243	−.0004	(0.008)	.955	−.002	(0.005)	.609
C												
BMIC (μg L^−1^)	.001	(0.001)	.105	.003	0.001)	.013	−.0006	(0.001)	.600	−.0003	(0.0007)	.724
Leptin (ng ml^−1^)	.131	(0.152)	.391	.322	(0.205)	.119	−.195	(0.209)	.353	.136	(0.131)	.301

*Note*: Adjusted for maternal age, educational level, pre‐pregnancy maternal body mass index, parity, type of feeding at 6 months and anthropometric measures at birth.

Abbreviations: BMIC, breast milk iodine concentration; IGF‐1, insulin‐like growth factor‐1.

## 4 DISCUSSION

In this study, we assessed the associations between iodine and growth‐ and obesity‐related hormones in breast milk and subsequently infants' growth measurements. No associations were observed between iodine and IGF‐1, AD or LP in breast milk in the early stage of lactation. In our study, higher concentration of breast milk iodine was associated with higher infant weight‐for‐length *z*‐score values at 6 months of life, whereas none of the breast milk hormones showed significant association with infants' anthropometric measures.

Although it is difficult to investigate how breastfeeding influences and regulates both short‐ and possibly long‐term growth, there are some studies indicating that breast milk composition might play an important role in determining infant growth (Lind, Larnkjaer, Molgaard, & Michaelsen, 2018). Of all the bioactive compounds identified in breast milk, AD and LP are the most common hormones studied with regard to growth (Fields et al., 2016; Mazzocchi et al., 2019). It has been shown that AD might have insulin‐sensitizing effects, and LP might induce satiety in the infants. Only few longitudinal studies investigated the possible association between these breast milk hormones during the early stages of lactation and infant growth status (Doneray, Orbak, & Yildiz, 2009; Miralles, Sanchez, Palou, & Pico, 2006). There is more consensus in the literature that higher total levels of breast milk AD were associated with lower weight or weight‐for‐length *z* scores in early infancy (<6 months) (Brunner et al., 2015; Chan et al., 2018; Woo et al., 2009; Yu et al., 2018). However, no definitive conclusion has yet been established regarding the exact role of LP in early body weight regulation. Some studies found negative associations between breast milk LP concentrations during the first weeks of the postpartum period and weight gain or BMI during infancy and early childhood (Miralles et al., 2006; Schuster, Hechler, Gebauer, Kiess, & Kratzsch, 2011), whereas others reported no association between breast milk LP at 6 weeks and infant outcomes (growth or body composition) later in life (Brunner et al., 2015). In our study, no association was observed between breast milk LP and AD levels at 3‐ to 5‐day postpartum and infants' anthropometric measures at 6 months of life. Another breast milk hormone that might be involved in the regulation and control of fetal growth and development is IGF‐1. Previous studies demonstrated that *serum* IGF‐1 levels were closely associated with body weight and length in infants (Savino et al., 2005; Wang, Xing, Qi, Guan, & Zhang, 2013); however, little is known about the effects of breast milk IGF‐1 on infant growth parameters. To the best of our knowledge, only one study has reported a positive association between breast milk IGF‐1 and infant growth and weight gain at 3 months of life (Kon, Shilina, Gmoshinskaya, & Ivanushkina, 2014). However, our finding showed that none of the anthropometric measures at 6 months of life were associated with breast milk IGF‐1 concentration.

Several factors could influence the levels of bioactive components in breast milk. In case of breast milk hormones, there is clear evidence that breast milk LP has been positively associated with maternal BMI (Fields et al., 2016). It has been also shown that maternal BMI, parity, length of gestation, race/ethnicity, type of delivery and smoking all contribute to the variation of AD in breast milk (Fields et al., 2016). Besides these factors, some recent studies propose that iodine in breast milk might potentially regulate the AD levels, although its underlying mechanisms remain unclear. In the study by Gutiérrez‐Repiso et al., a negative association between breast milk iodine and total levels of AD in lactating mothers was reported (Gutierrez‐Repiso et al., 2014). Consistently, in the study by Velasco et al., an inverse correlation between iodine and total AD in colostrum, but not in mature milk, was observed (Velasco et al., 2016). However, data indicating that iodine has a possible effect on LP or IGF‐1 levels in breast milk are not available. In the present study, breast milk iodine was not associated with growth‐ or obesity‐related hormones during the first few days of lactation. The interpretation of findings at this time may be difficult given the limited number of studies investigating these potential associations.

There is a strong biological basis regarding the role of iodine in somatic growth; however, available studies have reported inconsistent findings in different stages of life. For instance, in a recent systematic review and meta‐analysis by Nazeri et al., birth weight, length and head circumference were not associated with maternal iodine status during pregnancy, and infants born to iodine‐sufficient women did not have better growth parameters than infants born to iodine‐deficient women (Nazeri, Shab‐Bidar, Pearce, & Shariat, 2020). However, positive associations between both inadequate iodine intake and severe stunting and adequate iodine intake and appropriate growth in children have been clearly shown (Kramer, Kupka, Subramanian, & Vollmer, 2016). A similar pattern was also observed in a few observational studies investigating the association between postnatal iodine status and growth parameters. In a cross‐sectional study by Yang et al., the lowest mean values of head circumference‐for‐age and weight‐for‐age were observed in infants whose mothers had urinary iodine concentration less than 50 μg L^−1^ (Yang et al., 2017). Despite the best criteria for assessing an infant's iodine status have not been established due to the difficulty of sample collection for urinary iodine in this age group, there is sufficient data suggesting that the iodine status of infants could be extrapolated from either breast milk or maternal urinary iodine concentrations (Dold et al., 2017; Nazeri, Dalili, et al., 2018). In a pilot study by Ellsworth et al., BMIC at 2‐week postpartum was positively associated with infants' weight‐for‐age and weight‐for‐length over the first year of life (Ellsworth, McCaffery, Harman, Abbott, & Gregg, 2020). This finding was similar to our study with a different technique for BMIC measurement. In present study, we found that iodine concentration of breast milk at few days postpartum was within optimal range and had a potential effect on infants' weight‐for‐length at 6 months of life, although its effect size was small. This discrepancy is possibly explained by the fact that the mechanisms underlying the control of growth during each period are distinct: (1) fetal, where the predominant endocrine factors controlling growth are insulin and the IGFs; (2) infancy, where growth is mainly dependent upon nutrition; (3) childhood, where the growth hormone–IGF‐1 axis and thyroid hormone are considered the most important; and (4) puberty, where along with the growth hormone‐IGF‐1 axis the activation of the hypothalamic–pituitary‐gonadal axis to generate sex steroid secretion becomes vital to growth completion (Murray & Clayton, 2013).

To our knowledge, this is the first study that attempted to test the hypothesis whether BMIC through growth‐ and obesity‐related hormones affects infants' growth, specifically during the first 6 months of life when breast milk is the only main dietary source of iodine for breastfed infants. However, there are some limitations in the study that should be considered. First, a single breast milk sample was collected from each mother, which precluded assessing the variations of breast milk components over time. Second, we did not measure serum hormone levels in infants, although prior studies have demonstrated the association between hormones, such as AD, LP and ghrelin in breast milk and infant serum (Savino, Benetti, et al., 2012; Savino, Lupica, et al., 2012; Weyermann, Beermann, Brenner, & Rothenbacher, 2006). Third, infant growth was evaluated using anthropometric measures; measuring body composition using more precise methods may provide accurate data on the regulatory role of breast milk components in infants' growth. Lastly, these findings need to be interpreted cautiously because this observational study with small sample size was derived from a randomized clinical trial, which restricts our ability to draw definitive conclusion regarding cause–effect relationships and generalize to the population. Also, due to the small effect size, further studies with larger sample size are required to gain a better understanding of these associations.

In conclusion, our findings confirm that individual bioactive components of breast milk may regulate infants' growth status. In this study, BMIC during the early stage of lactation was positively associated with weight‐for‐length *z* scores in infants at 6 months of age, independent of IGF‐1, AD or LP concentrations in breast milk. To date, a limited number of studies have investigated the association between BMIC and postnatal growth outcomes; the underlying mechanism of how iodine in breast milk affects infant growth status, however, remains to be elucidated.

## CONFLICTS OF INTEREST

None of the authors has any personal or financial conflicts of interest.

## CONTRIBUTIONS

PN and ZT conceptualized and designed the study, carried out the statistical analyses and drafted the initial manuscript. MH contributed to the biochemical analyses. PM and FA critically reviewed the manuscript for important intellectual content and supervised the project. All authors have read and approved the final manuscript as submitted and agreed to be accountable for all aspects of the work.
